# Keeping time in the lamina terminalis: Novel oscillator properties of forebrain sensory circumventricular organs

**DOI:** 10.1096/fj.201901111R

**Published:** 2019-11-28

**Authors:** Rebecca C. Northeast, Lukasz Chrobok, Alun T. L. Hughes, Cheryl Petit, Hugh D. Piggins

**Affiliations:** ^1^ Faculty of Biology, Medicine, and Health University of Manchester Manchester UK; ^2^ Present address: Department of Neurophysiology and Chronobiology Institute of Zoology and Biomedical Research Jagiellonian University in Krakow Gronostajowa Street 9 Krakow 30‐387 Poland; ^3^ Present address: School of Natural Sciences and Psychology Liverpool John Moores University Liverpool UK; ^4^ Present address: School of Physiology, Pharmacology and Neuroscience Biomedical Sciences Building University of Bristol Bristol BS8 1TD UK

**Keywords:** circadian, circumventricular organ, fluid balance, OVLT, SFO, suprachiasmatic

## Abstract

Drinking behavior and osmotic regulatory mechanisms exhibit clear daily variation which is necessary for achieving the homeostatic osmolality. In mammals, the master clock in the brain's suprachiasmatic nuclei has long been held as the main driver of circadian (24 h) rhythms in physiology and behavior. However, rhythmic clock gene expression in other brain sites raises the possibility of local circadian control of neural activity and function. The subfornical organ (SFO) and the organum vasculosum laminae terminalis (OVLT) are two sensory circumventricular organs (sCVOs) that play key roles in the central control of thirst and water homeostasis, but the extent to which they are subject to intrinsic circadian control remains undefined. Using a combination of ex vivo bioluminescence and in vivo gene expression, we report for the first time that the SFO contains an unexpectedly robust autonomous clock with unusual spatiotemporal characteristics in core and noncore clock gene expression. Furthermore, putative single‐cell oscillators in the SFO and OVLT are strongly rhythmic and require action potential‐dependent communication to maintain synchrony. Our results reveal that these thirst‐controlling sCVOs possess intrinsic circadian timekeeping properties and raise the possibility that these contribute to daily regulation of drinking behavior.

**Abbreviations**
OVLT: organum vasculosum laminae terminalis
SCN: suprachiasmatic nuclei
sCVOs: sensory circumventricular organs
SFO: subfornical organ
TTFL: transcriptional‐translational feedback loop
TTx: tetrodotoxin

## 1 INTRODUCTION

Robust daily rhythms of physiology and behavior are crucial for optimal health and well‐being.^1^, ^2^, ^3^ In mammals, intrinsic near 24 hours or circadian rhythms are driven by the master clock in the brain's suprachiasmatic nuclei (SCN). The retina directly innervates the SCN and this enables the entrainment of circadian rhythms to the external light‐dark cycle.^4^, ^5^ Individual SCN neurons rhythmically express core clock genes such as *Period1‐2* and function as autonomous clock cells, but require the intercellular signaling to synchronize and convey coherent circadian phase information to the rest of the brain and body.^6^

Rhythmic core clock gene expression occurs in the brain areas outside of the SCN, such as the olfactory bulb, mediobasal hypothalamus and the habenula, raising the possibility that circadian control of neural function is locally devolved.^7^, ^8^, ^9^, ^10^, ^11^, ^12^, ^13^ The generation of animals bearing clock gene reporter constructs, in particular the PERIOD2::LUCIFERASE (PER2::LUC) mouse, has enabled the real‐time visualization of clock gene oscillations ex vivo to study tissue‐level spatiotemporal dynamics as well as the behavior of single‐cell oscillators and their interactions.^14^, ^15^ Importantly, visualization of bioluminescence signals allows the investigation of potential timekeeping in smaller brain areas that would otherwise be undetectable using nonimage‐based luminometry.

Similar to many homeostatic processes, drinking behavior demonstrates clear circadian variation.^16^ Two key brain areas known for their roles in the central control of thirst homeostasis are the subfornical organ (SFO) and the organum vasculosum laminae terminalis (OVLT).^17^, ^18^, ^19^ These are sensory circumventricular organs (sCVOs); midline structures along the third ventricle characterized by their lack of a blood‐brain barrier and extensive vascularization.^20^ The SFO and OVLT respond to fluid balance signals and plasma hypertonicity to drive thirst‐related neural pathways and alter drinking behavior.^21^, ^22^ The SCN has direct neural connections with both the SFO and OVLT,^23^ and vasopressin neurons of the SCN drive nocturnal anticipatory thirst through excitation of OVLT neurons.^24^ Despite these clear observations of circadian variation in drinking behavior, there are currently no reports of endogenous circadian activity in the SFO, while circadian oscillations in the OVLT are not extensively defined.^25^, ^26^, ^27^

Here, we used ex vivo PER2::LUC bioluminescence imaging and in vivo gene expression to broadly assess the circadian rhythmicity of the SFO and OVLT. We provide the first description of a robust circadian clock in the SFO and compare its single‐cell oscillator properties to those of cells in the OVLT and the SCN. Using pharmacological and mechanical manipulations, we report unexpected characteristics to the maintenance of these rhythms. In addition, a comprehensive screening of circadian‐related genes in the SFO reveals robust 24 hours variation in molecular expression in vivo. Collectively, our results show the SFO and OVLT to possess the intrinsic timekeeping capabilities at whole tissue and individual cellular level.

## 2 METHODS

### 2.1 Animals

Mice were housed under 12:12 hours light‐dark conditions unless stated otherwise, with food and water available ad libitum. Mice used for bioluminescence and immunohistochemistry experiments were bred in‐house by the University of Manchester Biological Services Facility, except for qPCR studies for which C57BL6J mice were used (provided by Charles River, Kent, UK). All experiments and procedures were carried out in keeping with the UK Animal (Scientific Procedures) Act 1986 and with approval from the Research Ethics committee of the University of Manchester.

### 2.2 Bioluminescence imaging

#### 2.2.1 Culture preparation

For all bioluminescence imaging experiments, adult male PER2::LUC mice^15^ aged 10‐24 weeks were used. Animals were culled between ZT2‐4 (where ZT0 is lights‐on) and the brains were carefully extracted and immediately submerged in ice‐cold Hanks Balanced Salt Solution (HBSS; Sigma, Poole, UK) supplemented with 0.01 M HEPES (Sigma) and 1 mg/mL penicillin‐streptomycin (Gibco Invitrogen Ltd, Paisley, UK). For coronal slices, brains were mounted onto the stage of the vibroslicer (Campden Instruments, Leicester, UK) in ice‐cold HBSS and cut into 250 µm thick slices. Coronal slices containing maximal bioluminescence for the OVLT were located from 0.62 to 0.38 mm from the bregma, and −0.46 to −0.82 mm from the bregma for the SFO. Sagittal slices of the SFO were taken from −0.10 to 0.15 mm from lateral (sagittal suture). Coronal SCN slices were taken from −0.35 to −0.46 mm to the bregma. Anatomical coordinates from Paxinos and Franklin (2001) were used to assist with the dissections. The brain areas of interest were excised with a scalpel and explants placed on sterile culture inserts (Millipore Ltd, Watford, UK) in 35 mm culture dishes (Fluorodish, World Precision Instruments Ltd, Stevenage, UK). Culture dishes contained 1.4 mL of sterile recording media composed of DMEM; Dulbecco's modified Eagle's medium (D‐2902, Sigma) supplemented with 3.5 g/L D‐glucose (Sigma), 1 mg/mL penicillin‐streptomycin (Gibco), B27 (Invitrogen), 0.035% sodium bicarbonate (Sigma), 10 mM HEPES buffer (Sigma), and 0.1 mM luciferin, and were sealed with vacuum grease (Dow Corning Ltd, Coventry, UK) and a glass coverslip.

### 2.3 Forskolin and tetrodotoxin treatments

Forskolin (10 µM; Sigma) treatments were performed as fresh media changes on day 5 or day 7 of the recordings at CT6. Tetrodoxin (TTx; Tocris, Bristol, UK) experiments were performed with 0.5 µM TTx in the media for the duration of the recordings.

### 2.4 Data acquisition and analysis

Cultures prepared as above were immediately transferred to the heated stage (37°C) of the bioluminescent imaging system Luminoview LV200 (Olympus, Japan) fitted with a cooled Hamamatsu ImageEM C9100‐13 EM‐CCD camera and a 20 x 0.4 NA Plan Apo objective (Olympus). Darkness was maintained throughout the recordings and exposure time was 60 minutes for OVLT and SFO and 30 minutes for SCN. Camera gain was the same for OVLT and SFO, but due to the intensity of the signal, it was lower for SCN recordings. Images were analyzed in ImageJ, using an ROI selection tool to outline the putative single cells or whole brain areas for measuring relative bioluminescence over time. Raw data were subject to a 3‐hours running average smooth and the first 12 hours of all recordings were excluded prior to analysis. Peaks and troughs of individual bioluminescence traces were determined manually. At least three peak‐to‐peak and two peak‐to‐trough measurements were used to calculate the period and amplitude, respectively. Damping rate was determined as relative to the amplitude of the peak on day 2 for baseline or day 0 of forskolin treatment. Rayleigh plots of peak phase and their corresponding r values were created using El Temps (University of Barcelona, Spain).

### 2.5 Statistics

Analysis of variance (with Tukey's or Sidak posthoc tests) and *t* tests (or Mann‐Whitney U tests) were used to compare the parameters of explants rhythms across the different structures. All statistical analyses were performed using Prism 7 (GraphPad Software, USA). Unless stated otherwise, data are presented as mean ± SEM.

### 2.6 Quantitative Real‐Time PCR

#### 2.6.1 Tissue preparation and RNA extraction

Ten‐week‐old male C57BL6J mice (n = 19) were placed into constant darkness for 36‐48 hours and culled at four time points corresponding to the circadian time (CT) 0, 6, 12, and 18 (n = 4‐5 mice/CT). Brains were removed and flash frozen in dry ice. About 20 µm sections containing the SFO were cut onto PEN‐membrane slides (Leica Biosystems, Germany) using a cryostat (Leica CM3050 S). For laser‐capture microdissection (LCM), sections were stained with 1% cresyl violet (Sigma) in 70% ethanol before regions of interest were extracted on a laser‐capture microscope system (Leica DM6000 B) and stored in lysis buffer (Promega). Subsequently, RNA was extracted from the dissected tissue using the ReliaPrep RNA Tissue Miniprep System (Qiagen, USA). Reverse‐transcription was performed using the High‐Capacity RNA‐to‐cDNA Kit (Applied Biosystems, USA).

### 2.7 RT^2^ Profiler PCR Array

Extracted cDNA was then amplified using the RT2 PreAMP Pathway Primer Mix and the RT2 PreAMP PCR Mastermix (catalog number: PBM‐153Z, Qiagen, UK) in accordance with the manufacturers protocol. The resulting product was then loaded onto a RT^2^ Profiler PCR Array (catalog number: PAMM‐153Z), a PCR plate preloaded with qPCR primers of 84 genes related to circadian rhythms (Supplemental Table S1). Thermal cycling and data collection were performed using Applied Biosystems 7900HT Fast Real‐Time PCR.

### 2.8 Data and statistical analysis

Data analysis was performed in accordance with the manufacturer's guidelines using the ΔΔCT method, whereby GAPDH was used as the housekeeping gene with CT0 values used as the relative target gene expression. Due to the non‐Gaussian distribution of values in most groups, the Kruskal‐Wallis test was used to assess the temporal variation in gene expression. All data are shown as means for each time point ± SEM.

### 2.9 Immunohistochemistry

#### 2.9.1 Tissue preparation

*Per1*::Venus mice^28^ aged 10‐20 weeks (n = 4) were culled at ZT6 (to correspond with the high expression of *Per1*) by terminal injection of pentobarbital (80 mg/kg). Mice were then immediately transcardially perfused with ice‐cold 0.1 M phosphate‐buffered saline (PBS) followed by 4% solution of ice‐cold paraformaldehyde (PFA) in PBS. Following excision, brains were then postfixed in the 4% PFA in PBS overnight in 4°C. Brain tissue was then cryoprotected in 30% w/v solution of sucrose in PBS. Brains were then sliced into 35 µm thick coronal slices using a freezing sledge microtome (Bright Instruments, UK).

### 2.10 Immunohistochemical staining and imaging

The desired brain sections containing the SFO and OVLT were initially washed free‐floating in PBS. Next, brain tissue was permeabilized with 0.1% Triton X100 (Sigma) for 20 minutes at room temperature (RT), following further washing in PBS. Slices were then blocked in 5% normal donkey serum (NDS; Sigma) and 0.05% Triton X100 at RT for 30 minutes. A 48 hours incubation at 4°C in a PBS solution containing 0.05% Triton X100, 0.5% NDS and the primary antibodies rabbit anti‐GFP (1:1000, Abcam), and chicken anti‐vimentin (1:4000, Abcam). Following this incubation, slices were washed in PBS and transferred to PBS solution of secondary antibodies for 24 hours at 4°C; donkey anti‐rabbit Cy5 (1:800, Jackson ImmunoResearch) and donkey anti‐chicken Alexa 488 (1:800, Abcam). The secondary antibodies were then washed off in PBS and the slices were mounted on gelatine‐coated glass slides using VectaShield medium (Vector Laboratories, USA). Slices were then imaged on a confocal microscope (Leica SP5 upright) under the 20x objective. Z‐stacks were acquired in 3 µm steps and viewed as maximal intensity projections.

## 3 RESULTS

### 3.1 The SFO and OVLT exhibit robust oscillations in PER2::LUC

**Figure 1:**
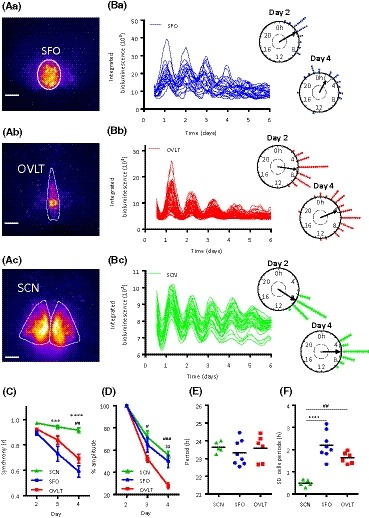
Circadian rhythms in the SFO and OVLT. False‐colored representative image of bioluminescence expression in the (Aa) SFO, (Ab) OVLT, and (Ac) SCN of coronal brain slice explants, where warmer colors indicate higher expression. White bars depict 100 µm. Note the increased density of putative single cells in the SFO vs the OVLT. Example traces of putative single‐cell bioluminescence from the (Ba) SFO, (Bb) OVLT, and (Bc) SCN, and their corresponding Rayleigh plots showing peak phase clustering of these individual cellular oscillations on day 2 and day 4 of the recording. Circles represent peak bioluminescent phases for individual cells (blue = SFO; red = OVLT; green = SCN), the arrow indicates the phase vector and the arrow length represents significance with respect to the inner dashed circle detailing the (*P* = .05) significance threshold. Variance is represented by the black box adjacent to the arrow head. C, Phase clustering of individual cells in the SCN, SFO and OVLT for peaks 2‐4 (calculated using the Rayleigh *r* value); the SCN is more synchronized than the SFO on days 3 and 4 and more synchronized than the OVLT on day 4. Two‐way RM ANOVA, Tukey's multiple comparisons test SCN vs SFO ***P < .001 ***P < .0001, SCN vs OVLT^##^ *P* < .01. Data are mean ± SEM. D, Damping (relative to the amplitude of the peak in day 2) is reduced in the OVLT vs the SCN on days 3 and 4, and reduced in the OVLT vs SFO on day 4. Two‐way RM ANOVA, Tukey's multiple comparisons test, SCN vs OVLT ^#^ *P* < .05 ^###^ *P* < .001, SFO vs OVLT $$*P* < .01. Data are means ± SEM. E, The period of single‐cell oscillations does not differ between the SCN, SFO, and OVLT, F, but the standard deviation (SD) of these individual cellular periods is reduced in the SCN compared with the SFO and OVLT. One‐way ANOVA, Tukey's multiple comparisons test SCN vs SFO ****P < .0001. SCN vs OVLT ^##^ *P* < .01. Black horizontal lines represent the mean value. RM = repeated measures

Evidence of intrinsic circadian rhythms in sCVOs is unresolved.^25^, ^27^ To address this gap in our knowledge, we used a sensitive imaging system and visualized PER2‐driven bioluminescence in coronal brain slices containing the SFO (Figure 1Aa) or OVLT (Figure 1Ab) or SCN (Figure 1Ac). PER2::LUC bioluminescent signal could be resolved to putative single‐cell oscillators, and tracked over 6 days of recordings in the SFO (Figure 1Ba) and OVLT (Figure 1Bb) and SCN (Figure 1Bc). It is well‐established that SCN cells remain highly synchronized^29^, ^30^ and to benchmark the intrinsic circadian timekeeping capabilities of the sCVO oscillators, we compared the circadian parameters of SFO and OVLT single cells with those of SCN cells recorded on the coronal plane (Figure 1Ac,Bc). Analysis of these revealed that within each structure, individual cells exhibited robust high‐amplitude circadian oscillations which were initially synchronized, but as shown through Rayleigh vector (*r*) analyses, they visibly desynchronized at a similar rate in sCVO structures (SFO: *r* slope = −.15 ± .006; OVLT: *r* slope = −.12 ± .018; *P* = .32), but not the SCN (*r* slope = −.03 ± .001) (Figure 1C). In comparison to the SCN (two‐way RM ANOVA, main effects and interaction, *P* < .05), the SFO was less synchronized on day 3 (Tukey, *P* = .0006) and day 4 (Tukey, *P* < .0001), while the OVLT was less synchronized than the SCN on day 4 (Tukey, *P* = .0006). This indicates that cells of both sCVO lack the ability of their SCN counterparts to maintain synchrony. The amplitude of whole area bioluminescence decreased in all structures (two‐way RM ANOVA significant, main effects and interaction, *P* < .05), and in comparison to the SCN, the relative amplitude in OVLT was less than that of SCN on day 3 (Tukey, *P* = .013) and day 4 (Tukey, *P* = .0003), and less than that of the SFO on day 4 (Tukey, *P* = .0013) (Figure 1D). This indicates that SFO oscillators are better able to maintain their amplitude over time than OVLT oscillators. The mean periods of the single‐cell oscillators were similar in all structures (SFO 23.31 ± 0.25 h, OVLT 23.58 ± 0.30 h; SCN 23.31 ± 0.25 h; all comparisons *P* > .05) (Figure 1E), but compared to the SCN, single‐cell periods were much more variable (one‐way ANOVA, *P* < .0001) in the SFO and OVLT (SCN vs SFO Tukey *P* < .0001; SCN vs OVLT Tukey *P* < .01) (Figure 1F). This analysis reveals that within tissue explants, SCN clock cells are better able to maintain synchrony and are more precise in their periodicity than their counterparts in the SFO or OVLT.

### 3.2 In vivo rhythmic gene transcript expression in the SFO

**Figure 2:**
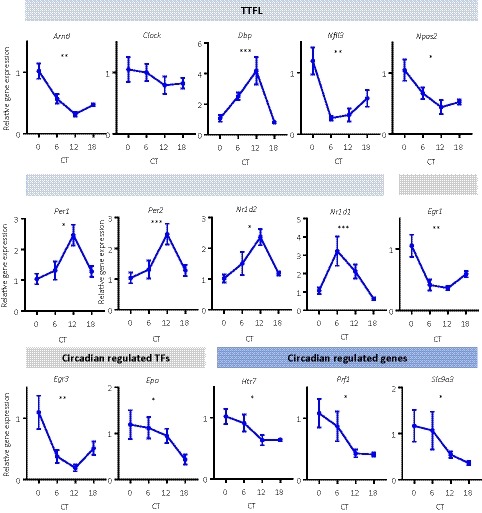
In vivo expression shows temporal variation in the SFO. Temporal variation of genes related to the generation and maintenance of circadian rhythms sampled every 6 hours across the circadian cycle, relative to CT0. Kruskal‐Wallis test, *P < .05, **P < .01, ***P < .001. TTFL = transcriptional translational feedback loop, TFs = transcription factors. Data are plotted as mean ± SEM

The above investigations indicate that, ex vivo, coronal brain slice explants of SFO and OVLT exhibit circadian oscillations in clock gene expression. To investigate whether circadian clock gene expression in an exemplar sCVO varied over 24 hours in vivo, we placed mice in constant dark and sampled the SFO every 6 hours across the circadian cycle, starting at CT0 (Supplemental Table S1). Subsequently we used a qPCR plate preloaded with genes related to circadian rhythms and determined if and how these genes changed in their expression in the SFO over the circadian cycle. We discovered that 8 out of 16 circadian clock gene transcripts involved in the transcriptional‐translational feedback loop (TTFL)^31^ showed significant variation over time. These were *Arntl* (BMAL1; *P* < .01), *Dbp* (DBP, D‐box binding protein; *P* < .001), *Per1* (PER1; *P* < .05), *Per2* (PER2; *P* < .001), *Nfil3* (NFIL3, nuclear factor interleukin‐3 regulated; *P* < .01), *Nr1d1* (Rev‐erbα; *P* < .001), *Nr1d2* (Rev‐erbβ; *P* < .05), and *Npas2* (NPAS2; *P* < .05) (Figure 2). Expression of *Dbp*, *Nr1d2*, *Per1*, and *Per2* was high around CT12, whereas the expression of *Arntl*, *Nfil3*, and *Npas2* was in near antiphase, approaching maximal levels around CT0. The other transcripts demonstrating significant temporal variation included three circadian‐regulated transcription factors *Egr1* (EGR1, early growth response 1; *P* < .01), *Egr3* (EGR3; *P* < .01), and *Epo* (EPO, erythropoietin; *P* < .05), and three common circadian‐regulated genes *Htr7* (5‐HT7, 5‐hydroxytryptamine receptor 7; *P* < .05), *Prf1* (PRF1, perforin 1; *P* < .01), and *Slc9a3* (NH3E, sodium hydrogen exchanger 3, *P* < .05) (Figure 2). These data provide further evidence of circadian control of the SFO in vivo and allude to processes that this clock may regulate.

Furthermore, to visualize clock gene expression, in vivo we performed immunohistochemistry on coronal brain sections containing the SFO or the OVLT from *Per1*::Venus fluorescent reporter mice (Figure S1). Immunostaining for vimentin was used to delineate the glial processes and to define the borders of the SFO and OVLT.^32^ In both sCVOs, *Per1* + ve nuclei were numerous and in close proximity to glial processes. However, unlike the SFO in which *Per1* + ve nuclei were mostly contained within its anatomical boundaries, at the level of the OVLT, many *Per1* + ve nuclei were visible in the adjacent medial preoptic area where rhythms in PER2::LUC are absent. This indicates that the medial preoptic area lacks the intrinsic circadian timekeeping properties of the OVLT.

### 3.3 Forskolin treatment re‐synchronizes individual cellular oscillations and generates long‐lasting whole tissue rhythms in the SFO and OVLT

**Figure 3:**
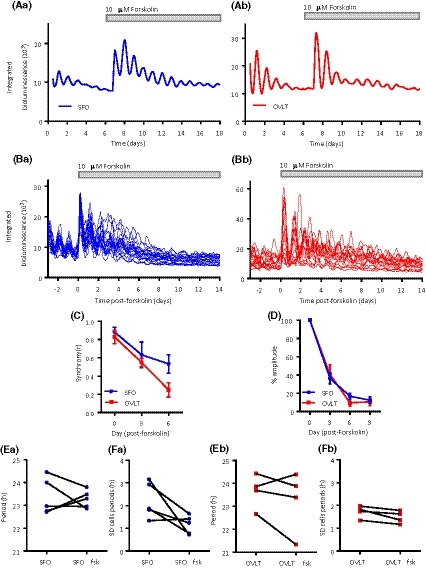
Forskolin promotes long‐term rhythms in PER2::LUC expression. Whole area bioluminescence rhythms prior to and following forskolin treatment for the (Aa) SFO and (Ab) OVLT, demonstrating long‐lasting synchrony and clear oscillations up to 14 days post‐forskolin treatment. Individual cellular rhythms revived and synchronized post‐forskolin treatment in the (Ba) SFO and (Bb) OVLT. C, Phase clustering of individual cells post‐forskolin treatment; synchrony did not vary between the structures. D, Damping rate relative to the first peak after forskolin treatment (day 0) showed no change between the structures. E, The mean period of single‐cell oscillations did not change pre‐ and post‐forskolin treatment, nor did change the F, variability in these individual oscillator periods. Duration of forskolin treatment is denoted by the gray‐filled bar. Data in C and D plotted as mean ± SEM

The adenylate cyclase activator forskolin resets and resynchronizes cellular and whole tissue rhythms of bioluminescence in the SCN and many extra‐SCN areas.^10^ To assess if sCVO rhythms are similarly reset, forskolin (10 µM) was applied to SFO and OVLT explants whose PER2::LUC rhythms had visibly damped following 7 days in culture. Consistent with its actions in the SCN and other extra‐SCN oscillators,^33^ treatment with forskolin induced high‐amplitude oscillations which subsequently decreased in relative amplitude but remained clearly rhythmic for over 14 days posttreatment (n = 5 SFO; n = 4 OVLT) (Figure 3A). Similarly, individual cells whose rhythms had become damped and desynchronized were strongly resynchronized by forskolin, remaining rhythmic for up to 6 days post‐forskolin treatment, after which peaks and troughs from individual cells became difficult to reliably distinguish (Figure 3B,C). No significant difference was observed in the rate of desynchrony between the SFO and OVLT (SFO: *r* slope = −0.06 ± 0.014; OVLT: *r* slope = −0.10 ± 0.002, *P* = .067) with the SFO tending to decrease at a slower rate. Relative bioluminescence damping did not vary over 9 days following forskolin treatment (Figure 3D). In the SFO and OVLT, the average periods of single‐cell oscillations did not change from pre‐ to post‐forskolin treatment (SFO: 23.38 ± 0.80 h vs 23.28 ± 0.39 h; OVLT: 23.66 ± 0.74 h vs 23.25 ± 0.67 h) (Figure 3E). Forskolin tended to decrease the variability in the single‐cell oscillator periods in the SFO (SD; pre‐ 2.22 ± 0.78 vs post‐forskolin 1.16 ± 1.16 h, *P* = .06) (Figure 3Fa), but not in the OVLT (SD; pre‐ 1.72 ± 0.27 vs post‐forskolin 1.48 ± 0.27 h, *P* > .75) (Figure 3Fb).

### 3.4 Tetrodotoxin treatment abolishes single‐cell oscillator synchrony

**Figure 4:**
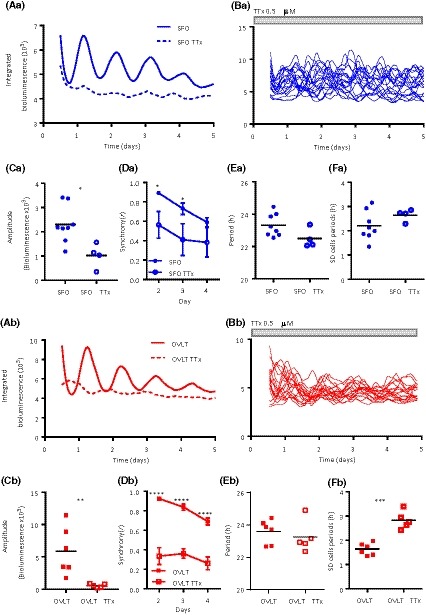
Tetrodotoxin treatment decreases single‐cell synchrony. Representative mean traces of individually oscillating single cells recorded during control baseline and in the presence of TTx for the (Aa) SFO and (Ab) OVLT and their corresponding individual cell traces (Ba; SFO, Bb; OVLT) during the TTx recordings, grey‐filled bar indicates TTx duration. Note that while individual cells continue to oscillate, they are not in synchrony with one another. Whole area bioluminescence amplitude is significantly decreased for control baseline vs TTx treatment in the (Ca) SFO (*t* test *P < .05) and the (Cb) OVLT (Unpaired *t* test *P < .05). TTx treatment decreases the synchrony of individually oscillating cells during days 2 and 3 of the recording in the (Da) SFO and (Db) days 2‐4 in the OVLT. Two‐way RM ANOVA, Sidak's multiple comparisons test *P < .05, ****P < .0001. Data are means ± SEM. The mean period of the single‐cell oscillations is not affected by TTx treatment in the (Ea) SFO and (Eb) OVLT, with the (F) variability in these cellular periods being greater in the (Fb) OVLT. Unpaired *t* test, ***P < .001. Duration of TTx treatment is denoted by the gray‐filled bar. Black horizontal lines represent the mean value. RM = repeated measures. TTx = tetrodotoxin

Tetrodotoxin (TTx) is a voltage‐gated sodium channel inhibitor and thus prevents the action potential‐dependent communication between neurons. In SCN cultures, TTx reduces the amplitude of clock gene oscillation and synchrony among single SCN neurons.^34^, ^35^ To assess whether circadian PER2::LUC oscillations in the SFO and OLVT also require sodium channel‐dependent action potential mechanisms, we cultured coronal explants of the SFO and OVLT in the presence of 0.5 µM TTx. In comparison to baseline (TTx‐free) conditions, treatment with TTx significantly damped whole area bioluminescence rhythms (Figure 4Aa,Ab) in the SFO (*P* < .05) and OVLT (*P* < .05) (Figure 4Ca,Cb). Single cells in both sCVOs sustained rhythms in the presence of TTx (Figure 4Ba,Bb and Figure S2), but were unable to remain synchronized (SFO: two‐way RM ANOVA, main effects *P* < .05; OVLT: two‐way RM ANOVA, main effects *P* < .05). Compared to baseline conditions, single‐cell oscillations in the SFO were significantly less synchronized on days 2 and 3 (Sidaks, both *P* < .05) (Figure 4Da), while there was a small effect of TTx on cellular period (SFO: 23.31 ± 0.25 h vs 22.49 ± 0.30 h, *P* = .074; Figure 4Ea), but not the variability in these periods (*P* = .19; Figure 4Fa). Cells in the OVLT were significantly desynchronized by TTx treatment for days 2, 3, and 4 (all days, Sidaks, *P* < .0001; Figure 4Db), while the mean cellular period was unaffected (OVLT: 23.58 ± 0.74 h vs 23.24 ± 0.43 h, *P* = .53; Figure 4Eb). However, in contrast to the SFO, TTx increased the variability in OVLT cellular periods (*P* < .001; Figure 4Fb). This indicates that individual cellular oscillations in the OVLT are more reliant on action potential‐dependent neuronal communication than SFO cells.

### 3.5 SFO oscillations are not synchronized longitudinally

**Figure 5:**
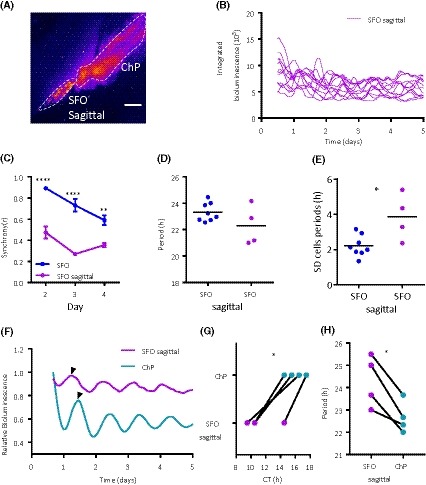
Imaging on the sagittal plane reveals longitudinal desynchrony in the SFO. A, False‐colored image of PER2::LUC bioluminescence in the SFO from a sagittal brain slice. Note the high‐amplitude rhythm in bioluminescence in the caudally adjacent third ventricle choroid plexus (ChP). White bars depict 100 µm. B, Single‐cell bioluminescence traces from a sagittal SFO recording. C, Single‐cell oscillations are desynchronized in recordings from sagittal SFO brain slice explants in comparison to coronal SFO recordings. Two‐way RM ANOVA (factors “structure” and “day”), Sidak's multiple comparisons test **P < .01, ****P < .0001. Data are means ± SEM. D, The periods of the single‐cell oscillations in the sagittal SFO are the same as in the coronal SFO recordings but with increased (E) variability in these single‐cell periods. Unpaired *t* test, *P* < .05. Black horizontal lines represent the mean value. F, Whole area relative bioluminescence of example traces from one slice containing the sagittal SFO and ChP (third ventricle choroid plexus). Note the black arrows depicting peak bioluminescence with the sagittal SFO peaking before the ChP. G, The CT (circadian time) of peak bioluminescence in the sagittal SFO consistently peaks before the ChP. Paired *t* test, *P < .05. H, Periods of the whole structures for the sagittal SFO and the ChP; the ChP has a shorter period. Paired *t* test, *P < .05

The spatiotemporal profile of clock gene expression in the SCN varies on different anatomical planes,^36^, ^37^ indicating the heterogeneity in intrinsic rhythm generating properties as well as the importance of anatomical connections for rhythmic gene expression. To determine if this also occurs in the SFO, we made sagittal brain sections and imaged bioluminescent signals of the SFO (Figure 5A) and the immediately posterior third ventricle choroid plexus (ChP; a robust and independently rhythmic structure (as shown by Myung et al, 2018^27^)). On the sagittal plane, bioluminescent cells could be visualized and tracked in the SFO (Figure 5B and Figure S3). In comparison to the recordings of SFO made on the coronal plane (two‐way RM ANOVA, main effects and interaction, *P* < .05), these individual cellular oscillations were much less synchronized from the outset (day 2 and 3; Sidaks *P* < .0001, day 4; Sidaks *P* < .01) (Figure 5C). While the mean periods of the single‐cell oscillators did not vary between coronal and sagittal SFO explants (23.32 ± 0.25 vs 22.3 ± 0.75 h, *P* = .13) (Figure 5D), their variability was increased on the sagittal plane (SD; 2.21 ± 0.21 h vs 3.85 ± 0.67 h, *P* < .05) (Figure 5E). Additionally, the sagittal SFO did not maintain a stable phase relationship with the strongly rhythmic ChP (Figure 5F). Initially, PER2::LUC expression in the sagittal SFO peaks 4‐5 hours before that of the ChP (CT; 11.25 ± 1.10 vs 16.00 ± 1.30 h, *P* < .05) (Figure 5G), but since the sagittal SFO has a significantly longer period than that of the ChP (24.3 ± 1.16 vs 22.67 ± 0.72 h, *P* < .05) (Figure 5H), the rhythms of these two structures drift further out of phase. These data reveal the spatiotemporal variation in timekeeping across the SFO and indicate that severing coronal anatomical connections within the SFO compromises synchrony between individual cell oscillations.

## 4 DISCUSSION

Our study provides evidence of robust circadian rhythmicity at both the whole area and single‐cell level in the SFO and OVLT, sCVOs implicated in central water homeostasis. Notably, we reveal for the first time the SFO to exhibit intrinsic timekeeping ex vivo, with core and noncore clock gene expression in this structure varying over the circadian cycle in vivo. Circadian rhythms in the SFO and OLVT are exceptionally sustained in culture, lasting up to 21 days following the treatment with forskolin. Moreover, similar to the SCN, rhythms in these sCVOs require action potential‐dependent communication to facilitate cellular synchrony. However, unlike the SCN, cells of the OVLT and SFO lack precision in their periodicities and cannot maintain synchronized rhythms. These properties of SFO single‐cell oscillators depend on the anatomical connections, as sectioning on the sagittal plane compromises their ability to maintain synchrony and lengthens their period.

Circadian rhythms in the OVLT have previously been demonstrated in clock gene bioluminescence^25^, ^26^, ^27^ and *Per3*.^38^ We extend these and are the first to demonstrate individual cell bioluminescence oscillations in the OVLT. These cells become desynchronized and reduced in amplitude over time, thereby contributing to the damping of whole area rhythms. To date, there have been no reports of circadian rhythmicity in the SFO, despite demonstration of its responsiveness to SCN output signals such as prokineticin 2 and vasopressin.^39^, ^40^, ^41^ The characteristics of individual cellular oscillations in the SFO were similar to those of the OVLT, and also to other extra‐SCN oscillators.^8^, ^10^ In comparison to the SCN, single‐cell oscillators in the SFO were less synchronized and displayed greater variability in their individual cellular periods, while the time of peak phase and average period of cells in these structures did not differ. Clock gene rhythms in extra‐SCN brain areas are often less robust than those of the master pacemaker,^9^, ^42^ thus it is surprising that whole area SFO oscillations were similar in relative amplitude over the first 4 days to the SCN. It is not clear why rhythms in the SFO are robust, but since this structure is enriched in the nonneuronal glia cells^32^, ^43^ and because astrocytes are important for circadian timekeeping in the SCN^44^, ^45^, ^46^, this network of nonneuronal cells may imbue the SFO with enhanced capability of self‐sustained circadian timekeeping. The SFO and OVLT also contain the specialized ependymal tanycytes^32^, ^43^ raising the possibility that multiple cell types could contribute to circadian rhythmicity in these structures. Furthermore, since these forebrain sCVOs lack a functional blood‐brain barrier, signals in the peripheral circulation may act to entrain and/or enhance their circadian properties.

Marked temporal variation in clock gene transcripts and other circadian‐regulated genes was readily quantified in the SFO in vivo. The core clock genes *Per1*, *Per2*, *Nr1d1* (*Rev‐erbα*), and *Nr1d2* (*Rev‐erbβ*) displayed a temporal variation, peaking approximately in antiphase with *Arntl* (*Bmal1*), as expected.^31^ This peak in *Per1‐2* appeared phase‐lagged compared to that seen in the SCN, suggesting a delay in the core molecular clock in the SFO.^47^ Interestingly, *Npas2*, a homolog of *Clock*,^48^ displayed significant variation over time with the absence of such variation seen in *Clock*, suggesting that *Npas2* is more dominant in the molecular clock in the SFO, which is also the case in the forebrain.^49^ The accessory core clock gene *Dbp* and its repressor *Nfil3* also displayed temporal variation in antiphase to each other, as seen in other tissues.^50^ Additionally, the transcription factors *Egr1* and *Egr3* displayed temporal variation. EGR1 regulates genes such as *Tnf‐α* and is involved in regulating the hepatic TTFL 51, 52 and both *Egr1* and *Egr3* expression are induced by light in the rodent SCN^53^, ^54^. This indicates that the SFO molecular clock contains functional accessory loops. In addition to mediating fluid balance, the SFO plays a role in immune function through the detection of circulating cytokines,^55^ and therefore temporal variation in the expression of the immune‐regulatory genes *Prf1* and *Nfil3* in the SFO is potentially of functional importance. Further regulatory roles of the SFO clock are suggested by temporal variation of *5‐HT7R* (*Htr7*) and NHE (*Slc9a3*) expression. The SFO is reciprocally connected with serotonergic neurons from the dorsal raphe nucleus^56^ and serotonin has been implicated in sodium appetite regulation,^47^, ^57^ for which the SFO is a key driver. NHE is a key sodium‐hydrogen exchanger whose expression is also rhythmic in the kidney,^58^ therefore it is intriguing that both these structures involved in water homeostasis display temporal regulation of this gene.

Forskolin reactivates and synchronizes damped cellular rhythms in the SFO and OVLT to produce further circadian oscillations for an unprecedented 21 days in culture. Studies of other extra‐SCN oscillators have yet to demonstrate such long‐term rhythms either spontaneously or following forskolin treatment.^8^, ^10^, ^25^, ^59^ This highlights the strikingly robust oscillatory properties of the SFO and OVLT and raises the possibility that they can function as autonomous circadian oscillators. Furthermore, since TTx damped their bioluminescent rhythms, this indicates that similar to the SCN,^34^, ^60^ intercellular synchrony in the SFO and OVLT requires action potential‐dependent communication. This is in contrast to some extra‐SCN oscillators, such as those in the mediobasal hypothalamus and lateral habenula, which still display individual cell and whole area rhythms in the presence of TTx.^8^, ^10^ The lack of damping displayed in those structures may be attributable to their rapid desynchrony during baseline conditions, making their dependency on action potential communication harder to determine. Alternatively, their rhythms are possibly generated through action potential‐independent mechanisms. Therefore, the SFO and OVLT display strong rhythmicity and rhythm maintenance properties that are similar to the SCN.

The SCN exhibits differential levels of clock gene expression across its rostro‐caudal axis suggesting spatiotemporal variation in timekeeping function and capability.^37^ Here we also find anatomical variation in timekeeping within the SFO. When sectioned on the sagittal plane, SFO cells are less able to maintain synchrony than when this structure is sectioned coronally. This indicates that anatomical connections preserved in the coronal plane but presumably not present on the sagittal plane are necessary for intercellular communication and synchrony. Furthermore, the clear difference in the time of peak PER2::LUC expression between the third ventricle ChP and sagittal SFO raises intriguing questions about their physiological relationship and the role of these autonomous clocks. One possibility for this change in phase relationship is the differing roles of these areas. The ChP produces cerebral spinal fluid, which is increased during the behaviorally quiescent phase and is an important contributor to the brain metabolite clearing process during sleep.^61^ In contrast, the SFO is involved in regulating thirst behavior, which is most prevalent during the behaviorally active phase.^19^ Therefore, the SFO clock peaking late in the day and the ChP clock peaking late in the night in mice could be preparing these structures for their primary functions.

**Figure 6:**
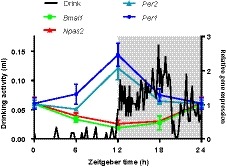
Expression of *Per1‐2* in the SFO is high at the onset of drinking activity. Schematic of relative in vivo gene expression of *Per1‐2*, *Bmal1*, and *Npas2* in the SFO (data from Figure 2), overlaid with 5‐day average 24‐hours drinking profile of a C57BL6/J male mouse. Gray shading represents lights‐off

Vasopressin neurons of the SCN directly innervate the OVLT to orchestrate the circadian regulation of late night anticipatory thirst which functions to prevent dehydration during the subsequent rest phase.^24^ An endogenous clock in the OVLT could allow the structure to anticipate rhythmic input from the SCN, enabling energy conservation during periods where certain mechanisms are in less demand. The SFO also receives input from SCN vasopressin neurons^23^ and has reciprocal connections with the SCN^56^ as well as with the OVLT that are necessary for the central control of thirst behavior.^17^, ^21^, ^62^ This raises the possibility that the SFO participates in anticipatory thirst and that its efferent signals feedback to the SCN as part of the circadian regulation of water homeostasis. In vivo *Per1* and *Per2* rhythms in the SFO peak at lights‐off, coincident with the onset of drinking activity in mice (Figure 6). Thus, the SFO may anticipate the night‐time increase in drinking associated signals or may even drive the nocturnal rise in fluid intake. Further studies are necessary to elucidate the relationship between clock gene expression and neuronal activity in these sCVOs.

In conclusion, our study characterizes robust circadian rhythms in the SFO and OVLT and that these possess intrinsic timekeeping properties that are much better sustained than previously reported in most other extra‐SCN oscillators. In their tissue networks, single‐cell oscillators in these structures lack the ability that SCN cells possess to cycle with precise periodicity and to maintain synchrony. We also show for the first time that genetic programmes within the SFO express overt variation in their expression over 24 hours. Altogether, our results highlight that these sCVOs are under more prominent circadian control than previous assumed, raising the possibility that local clock mechanisms in these structures contribute to daily regulation of thirst and fluid balance.

## AUTHOR CONTRIBUTIONS

RN, ATLH, LC, and HDP conceived the project and designed the bioluminescence studies. RN and ATLH conducted the bioluminescence studies, while RN and LC analyzed the data from these studies. RN and CP designed, conducted, and analyzed the in vivo gene expression study. RN, LC, and HDP wrote the manuscript. HDP supervised the project and provided financial support for the studies.

## CONFLICT OF INTEREST

The authors declare no conflicts of interest.
