# Supplementary material for: Keeping time in the lamina terminalis: Novel oscillator properties of forebrain sensory circumventricular organs
Source: FASEB J. 2019 Nov 28;34(1):974–87. doi: 10.1096/fj.201901111R (PMC6972491; doi:10.1096/fj.201901111R)
Supplement: Supplementary file 1 [file FSB2-34-974-s001.pptx]

## Slide 1
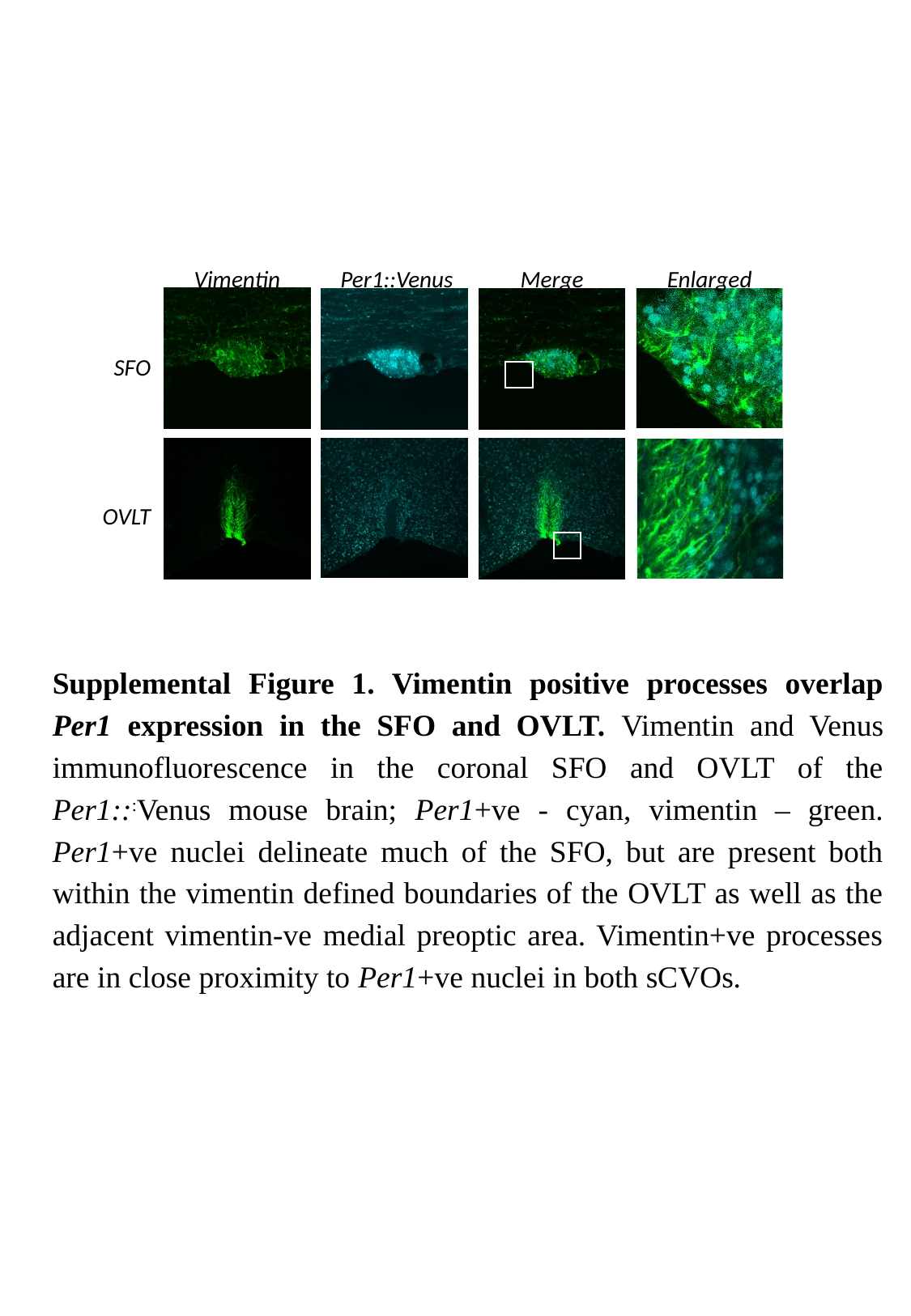

Per1::Venus
Enlarged
Vimentin
Merge
SFO
OVLT
Supplemental Figure 1. Vimentin positive processes overlap Per1 expression in the SFO and OVLT. Vimentin and Venus immunofluorescence in the coronal SFO and OVLT of the Per1:::Venus mouse brain; Per1+ve - cyan, vimentin – green. Per1+ve nuclei delineate much of the SFO, but are present both within the vimentin defined boundaries of the OVLT as well as the adjacent vimentin-ve medial preoptic area. Vimentin+ve processes are in close proximity to Per1+ve nuclei in both sCVOs.
