# Supplementary material for: Keeping time in the lamina terminalis: Novel oscillator properties of forebrain sensory circumventricular organs
Source: FASEB J. 2019 Nov 28;34(1):974–87. doi: 10.1096/fj.201901111R (PMC6972491; doi:10.1096/fj.201901111R)
Supplement: Supplementary file 2 [file FSB2-34-974-s002.pptx]

## Slide 1
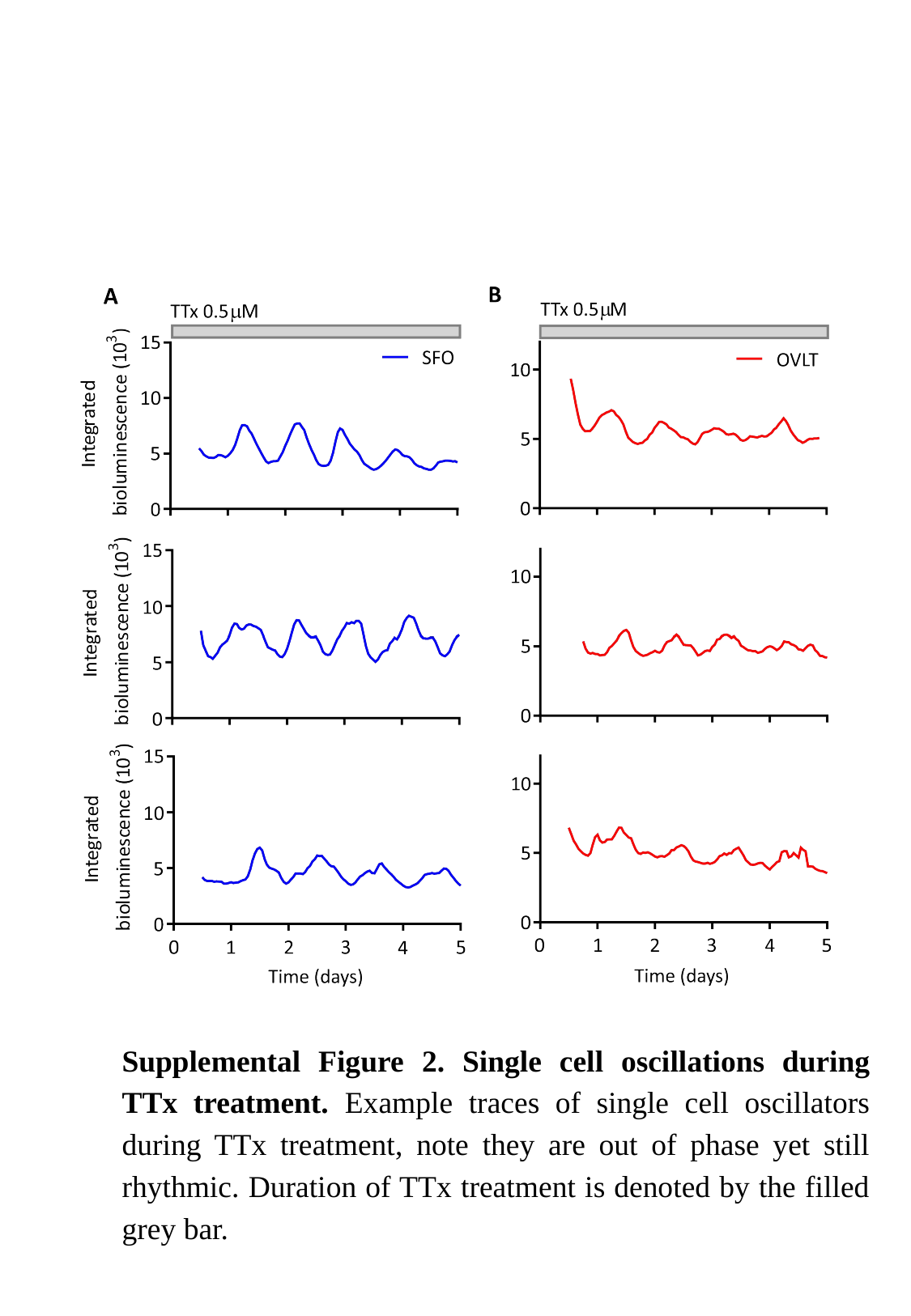

Supplemental Figure 2. Single cell oscillations during TTx treatment. Example traces of single cell oscillators during TTx treatment, note they are out of phase yet still rhythmic. Duration of TTx treatment is denoted by the filled grey bar.
