# Supplementary material for: Keeping time in the lamina terminalis: Novel oscillator properties of forebrain sensory circumventricular organs
Source: FASEB J. 2019 Nov 28;34(1):974–87. doi: 10.1096/fj.201901111R (PMC6972491; doi:10.1096/fj.201901111R)
Supplement: Supplementary file 3 [file FSB2-34-974-s003.pptx]

## Slide 1
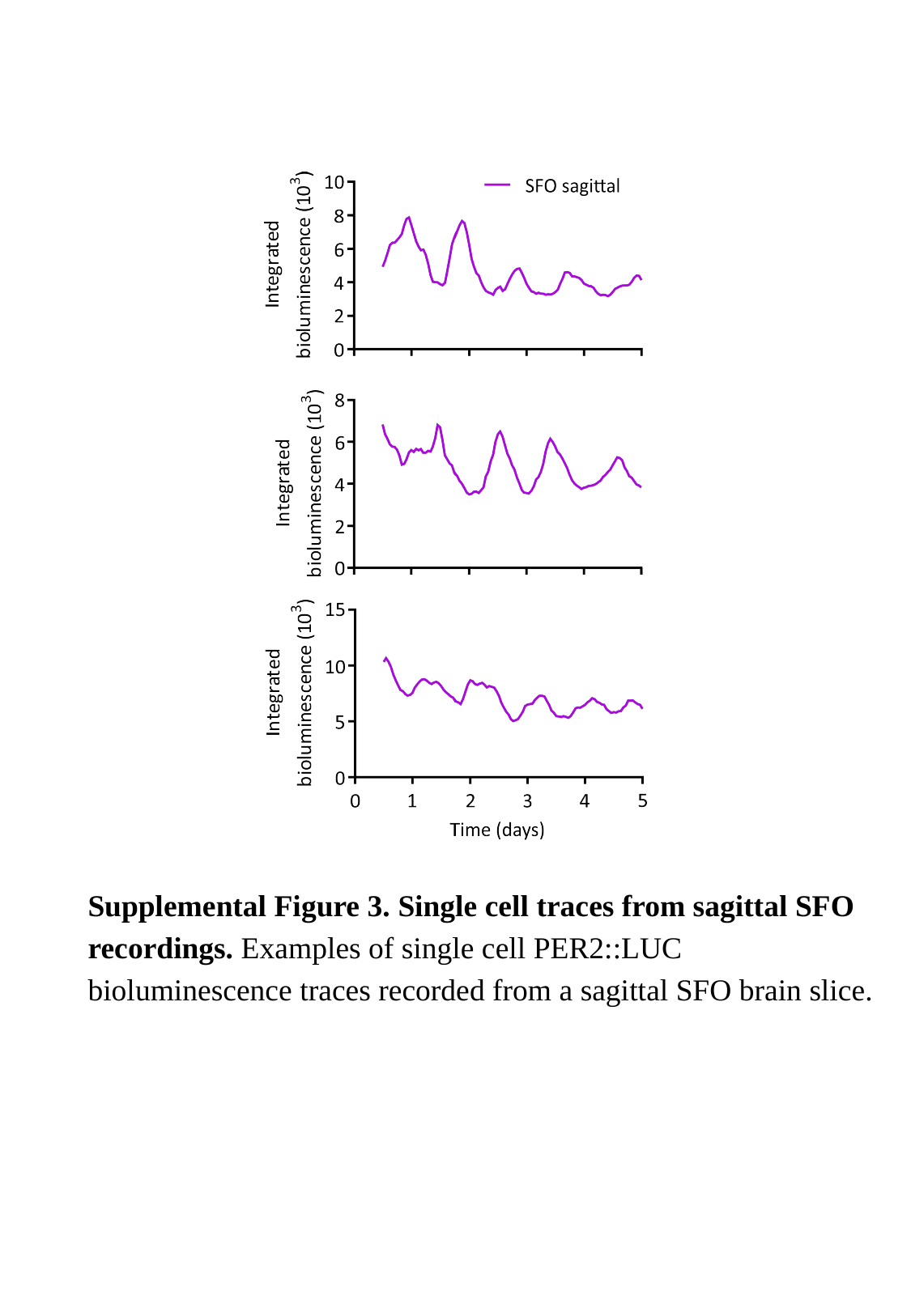

Supplemental Figure 3. Single cell traces from sagittal SFO recordings. Examples of single cell PER2::LUC bioluminescence traces recorded from a sagittal SFO brain slice.
