# Supplementary material for: Keeping time in the lamina terminalis: Novel oscillator properties of forebrain sensory circumventricular organs
Source: FASEB J. 2019 Nov 28;34(1):974–87. doi: 10.1096/fj.201901111R (PMC6972491; doi:10.1096/fj.201901111R)
Supplement: Supplementary file 4 [file FSB2-34-974-s004.docx]

|  | **Gene** | **P value** | **Summary** |  | **Gene** | **P value** | **Summary** |
| --- | --- | --- | --- | --- | --- | --- | --- |
| **TTFL** | Arntl | 0.0063 | ** | **Circadian** | Alas1 | 0.1043 |  |
|  | Arntl2 | 0.7434 |  | **regulated** | Egr1 | 0.0058 | ** |
|  | Bhlhe40 | 0.4104 |  | **TFs** | Egr3 | 0.0046 | ** |
|  | Bhlhe41 | 0.1435 |  |  | Epo | 0.0441 | * |
|  | Clock | 0.6078 |  |  | Esrra | 0.1299 |  |
|  | Cry1 | 0.9065 |  |  | Hlf | 0.2025 |  |
|  | Cry2 | 0.5378 |  |  | Irf1 | 0.726 |  |
|  | Dbp | 0.0002 | *** |  | Myod1 | 0.6387 |  |
|  | Nfil3 | 0.0096 | ** |  | Nkx2-5 | <dl |  |
|  | Npas2 | 0.0248 | * |  | Pax4 | 0.9415 |  |
|  | Nr1d1 | 0.0002 | *** |  | Pou2f | 0.2025 |  |
|  | Nr1d2 | 0.0354 | * |  | Ppara | 0.8007 |  |
|  | Per1 | 0.0213 | * |  | Smad4 | 0.1565 |  |
|  | Per2 | 0.0009 | *** |  | Sp1 | 0.9519 |  |
|  | Rora | 0.3812 |  |  | Srebf1 | 0.2219 |  |
|  | Rorb | 0.5867 |  |  | Stat5a | 0.3918 |  |
| **Circadian** | Cartp | 0.8309 |  |  | Tef | 0.5867 |  |
| **regulated** | Ccrn4l | 0.702 |  |  | Tfap2a | 0.0644 |  |
| **genes** | Fbxl21 | 0.1514 |  |  | Tgfb1 | 0.8771 |  |
|  | Fbxl3 | 0.289 |  |  | Wee1 | 0.0623 |  |
|  | Hebp1 | 0.0631 |  |  |  |  |  |
|  | Htr7 | 0.0137 | * |  |  |  |  |
|  | Ncoa3 | 0.0882 |  |  |  |  |  |
|  | Nms | 0.9415 |  |  |  |  |  |
|  | Nr2f6 | 0.8771 |  |  |  |  |  |
|  | Ppargc1a | 0.5553 |  |  |  |  |  |
|  | Prf1 | 0.0039 | ** |  |  |  |  |
|  | Ptgds | 0.7728 |  |  |  |  |  |
|  | Slc9a3 | 0.0401 | * |  |  |  |  |

**Supplemental Table 1.** Kruskal-Wallis test; light orange=p<0.09, light green/*=p<0.05, green/**=p<0.01, dark green/***=p<0.001. <dl=below detection limits. TTFL=transcriptional translational feedback loop, TFs=transcription factors.
